# The Associations between Father Involvement and Father–Daughter Relationship Quality on Girls’ Experience of Social Bullying Victimization

**DOI:** 10.3390/children9121976

**Published:** 2022-12-16

**Authors:** Shawndaya S. Thrasher, Esther K. Malm, Cana Kim

**Affiliations:** 1 School of Social Work, Louisiana State University, Baton Rouge, LA 70803, USA; 2 Department of Psychology, Murray State University, Murray, KY 42071, USA; 3 Department of Political Science, Louisiana State University, Baton Rouge, LA 70803, USA

**Keywords:** father involvement, father–child relationship quality, Black fathers, social bullying victimization, Fragile Families

## Abstract

With much research into physical, cyber, and verbal bullying victimization, social bullying victimization is a type of victimization that can be hidden. Studies about Black father involvement have found involvement to be a buffer to adverse and risky behaviors of children, including different forms of victimization experienced by their daughters. This study examined one gap in the literature: the direct and potentially indirect associations between father involvement and father–child relationship quality on child reports of social bullying victimization among girls. The cross-sectional sample of 368 Black fathers and their daughters was sourced from the Fragile Families and Child Wellbeing study. Data from wave 5 were selected for the child (age 9) and father because social bullying victimization was first measured at this time point. Logistic regression analysis findings showed father involvement was associated with lower social bullying victimization. In addition, talking and sharing ideas quite well rather than extremely well with their fathers was associated with higher odds of social bullying victimization. Father–daughter relationship quality did not mediate the father involvement and social bullying victimization relationship. Findings provide additional support to include fathers, particularly Black fathers, in intervention/prevention efforts and the importance of increasing awareness and benefits of father involvement in subtle forms of victimization such as social bullying victimization among Black families.

## 1. Introduction

Peer bullying victimization is a public health problem that negatively affects millions of children annually. Bullying may be direct or indirect and can manifest in several ways, including verbal, physical, social, and cyber forms [1]. Notably, social bullying victimization (SBV), which involves indirectly hurting one’s reputation or relationships (i.e., spreading rumors, being ridiculed by peers, and being left out/excluded from peer groups or activities), is identified as the most common and perhaps detrimental form of bullying [2]. SBV has been associated with an increased risk for emotional distress and mental health problems, especially for girls, who are more likely to be victims of social bullying, primarily in earlier years of development [3,4,5]. However, studies examining protective factors for social bullying victimization among girls during critical years of development, such as middle childhood, have been underexplored.

Parental involvement and healthy parent–child relationships can protect children from bullying victimization, though research has focused primarily on mothers alone [6,7,8,9]. Emergent research has, however, emphasized the essential role of fathers and father figures as a potential protective factor against their daughters’ experience of violence [10,11,12]. Yet this association is less explored for Black fathers in particular [10]. Studies that have examined Black father contributions, though limited to adolescent developmental periods so far, have concluded that their involvement and stronger father–daughter relationships are critical in reducing the number of adverse outcomes, especially those that disproportionally affect girls, such as risky sexual behaviors, unintended pregnancies, and placement in the juvenile legal system [13,14,15,16,17,18,19,20]. However, less is known about whether Black father involvement and the quality of the father–daughter relationship also serve as protective factors for bullying victimization, particularly SBV, a common form of victimization experienced among girls during middle childhood. The current study examined the direct and potentially indirect associations between father involvement and father–child relationship quality on child reports of SBV among girls.

### 1.1. Peer Bullying Victimization and Social Bullying Victimization (SBV)

Bullying victimization is the experience of unwanted, repetitive aggressive behavior among school-age children that involves a real or perceived power imbalance between the victim and perpetrator/bully [21]. The phrase “bullying victims” (not bully-victims) in this study specifically refers to victims of bullying behaviors in the school setting. Bullying victims experience physical, verbal, or social forms of aggression, which increase the risks of short and long-term outcomes such as poor psychopathology, unhealthy social relations, problem behaviors, and suicide [22]. While earlier research and school-based prevention practices have paid greater attention to the visible forms of bullying [23], social bullying is being more recognized as an important form of youth aggression, given its unique impact.

Social bullying victimization involves someone’s reputation or relationship being intentionally sabotaged instead of physical (i.e., hitting) or verbal (i.e., teasing) forms of peer bullying victimization. This involves direct and indirect physical and verbal relationship manipulations that lead social bullying victims to be disliked by peers, have fewer friends, and report higher levels of conflict within their friend groups [24,25,26]. Some studies describe these behaviors as relational bullying victimization [27,28]. The prevalence of SBV is large enough to go unnoticed and can result in negative consequences, including peer rejection, externalizing problems, depressive symptoms, low self-esteem, low self-efficacy, and loneliness [3,28,29]. Exposure to social bullying also increases long-term health problems such as depression and anxiety in young adulthood [27,30]

Social bullying is distinctive from other types of bullying (i.e., physical and verbal). First, since social bullying is subtle and neither easily observed nor the perpetrator easily identified, school personnel may be unaware of or have difficulty identifying the source of the conflict, thus failing to respond with proper actions [31,32]. Victims may also be less willing to report social bullying than other visible forms because they perceive that it should be addressed without adult involvement [33,34,35]. Since students experience social bullying under the surface, a deliberate and considerate approach to prevention strategies is urgently needed.

Second, social bullying is more widely experienced among girls than boys, and this trend is even more striking and higher in middle childhood [4,5,36]. For example, among youth aged 12–18, 3.5% of boys reported being excluded from activities on purpose compared to 6.9% of girls, while 9.3% of boys reported being the subject of social bullying, and about 17.5% of girls suffered from it [36]. In addition, a large-scale survey of students in grades 3–8 revealed that during a 4-week period nearly half of all girls reported social bullying victimization (41–48%) compared to 31–42% of boys [5]. Previous studies have also suggested that social bullying may impact girls more adversely than boys because they tend to place more value on relationships [37]. Accordingly, female social bullying victims have a higher risk of social anxiety, social avoidance, loneliness, feelings of distress, and behavioral problems [3].

Nevertheless, experiencing social bullying does not guarantee negative consequences. Family dynamics, such as involvement and relationship quality, are known to lower the risk of bullying victimization [9] and its consequences in general [38] and have been documented as one of the protective factors that may increase the resilience of a child experiencing bullying victimization [39]. Effective school-based bullying prevention programs have sought to involve families as a part of the whole-school approach [1,40], although few interventions have been implemented over the years.

### 1.2. The Significance of Father Involvement and the Father-Daughter Relationship

Parental involvement, defined as engagement or direct interaction with the child (i.e., helping with homework, talking to the child about the day, playing with the child, etc.), and parent–child relationship quality are critical for child development and have been conceptualized as essential for relationship sustainability between parent and child [41,42,43,44,45,46]. Theoretical frameworks such as attachment theory [46] and the parenting process model [45] highlight the importance of parent characteristics, parent–child bonding starting from birth [44,45,46] and the daily interactions between parent (caregiver) and child through parenting behaviors over time. Attachment, as an intense and enduring emotional bond, is rooted in the child’s need to ensure survival [46]. Belsky’s parenting process model outlines that parent characteristics and key external factors are determinants of parents’ well-being which influence parenting behaviors, and, in turn, directly and indirectly influence child outcomes [8,45]. Many studies focus on sections of the parenting process framework by testing direct, indirect, or concurrent effects over time and examine diverse parenting behaviors such as parental warmth, parent–child relationship quality, and involvement [8,47,48,49,50].

Guided by such theoretical frameworks, most parenting studies have focused almost exclusively on mother–child dyads [50]. However, over the years, and considerably among emergent research, studies have begun to examine and unpack the influence of the father–child relationship and father involvement on child outcomes [17,51,52,53]. Father involvement and the quality of the father–child relationship, such as frequent and high-quality interactions with the child, convey parental responsiveness to the child’s needs, further fostering a protective bond as children tend to feel a greater sense of safety. Since the attachment relationship with fathers is also socially oriented, daughters with good relationships and frequent interactions with their fathers might be less likely to suffer from social skill deficits. Thus, father’s frequent involvement and better father–daughter relationship quality may lower the chances of social bullying victimization. To this end, a burgeon of research has emphasized the dire need to investigate the sole role of fathers on child outcomes above and beyond maternal influences [28,42,54,55].

While studies highlight father involvement and relationship quality as essential and protective in their children’s lives, much more of such literature has focused on father–son relationships [17,42,52,56,57,58]. Notably, studies that have emphasized the unique role of the father on their daughters’ outcomes conclude that father relationships and involvement affect a variety of aspects of their daughters’ lives, from personality development, mental health, and stress response to social–emotional well-being [47,48,49,59,60,61]. For example, studies show that the quality of interactions between a child and the father helps regulate the child’s emotions more effectively regardless of the residency status of the father, and research has concluded that this effect was higher for girls than boys. Father involvement with daughter, and father–daughter relationship quality have also been associated with safe sex practices, positive behavioral and academic outcomes, career success, and healthy emotional coping, and for some, these associations persisted in adulthood [17,51,52].

### 1.3. The Significance of Black Father Involvement and Relationship with Their Daughters

There is evidence among other studies to suggest that Black fathers are similarly more involved with their children across a range of involvement activities irrespective of residency status [62], when compared to White and Hispanic fathers [63,64]. For example, a study using data from over 2000 participants in the Fragile Families dataset found that among nonresidential fathers, Black fathers were more likely to engage in activities with their children than Hispanic fathers, and they also shared responsibilities more frequently and exhibited more effective co-parenting practices than both White and Hispanic fathers [63]. Other studies have emphasized the role of Black fathers in keeping their children safe from community and neighborhood victimization [65,66], with one study focusing particularly on father–son dyads [65]. While Black fathers may struggle to provide financial support, research suggests they contribute to the family unit in other, non-financial ways such as childcare, child monitoring, and helping children with homework, which also shows a larger percentage of Black fathers compared to both Hispanic and White fathers assisted their co-residential children with homework every day during the course of a month [19,64,67,68].

Regardless, Black fathers have played an integral part in protecting their daughters from numerous adverse outcomes (i.e., academic disengagement, alcohol use), including those found to disproportionally affect girls (i.e., sexual decisions and risk-taking behaviors; psychological functioning) [14,15,16,17,18,19,20]. In fact, feeling close to their fathers had a greater impact on a multitude of outcomes for daughters than sons (i.e., academic achievement, career success, depression, externalizing behaviors, and marital happiness), and these relationships were evident in adulthood [69]. While studies continue to emphasize Black father involvement in healthy child development, research focusing on the parenting behaviors of Black fathers, including involvement and relationship quality, in their daughter’s bullying victimization risks is limited.

### 1.4. Black Fathers and Daughters: Relationship Quality and Involvement on Bullying Victimization

Much of the research on child bullying victimization suggests that parents are vital in preventing such experiences, though studies have focused extensively on parents in general [38,70,71] or mothers [6,7,8,9], primarily among older children, and in homogeneous samples of boys or heterogeneous samples [52,72,73]. The few studies focusing solely on or including fathers have concluded that fathers play an important role in decreasing their children’s experiences of bullying victimization [42,52,54]. For example, positive father–child relationship quality and father involvement were protective factors in homogenous samples of boys [52,74], in that their sons experienced less bullying victimization.

Studies using heterogeneous samples have concluded paternal care, but not monitoring, to be a protective factor against school bullying victimization [70,75]. Other studies conclude that ease of communication with the father, but not the child’s perception of their father’s awareness nor father’s parental monitoring, decreased African American children’s experiences of bullying victimization [42,54]. However, father involvement in general was not found to be significant in decreasing peer victimization for African American children, but satisfaction with family and parental/guardian support, in general, was associated with lower reports of bullying victimization [42]. While there is some evidence that father involvement and relationship quality is protective of child bullying victimization, less is known about whether this overarching pattern is evident for Black father involvement and father–daughter relationship quality on a common form of victimization frequently experienced among girls, SBV. 

Besides direct path models in many studies, others have investigated more complex developmental models, including the mediating role of fathers related to bullying victimization, although research has focused mostly on father parenting behaviors as a mechanism by which the relationship between bullying and its consequences may operate [42,52,72]. For example, emotional support from fathers was found to significantly mediate the relationship between bullying victimization and depressive symptoms over time [28]. Since the fathers’ relationship with their daughters is critical to their development and is protective against other adverse outcomes that uniquely impact girls, further research in SBV is warranted.

### 1.5. Purpose of the Present Study

While relatively few studies have explored the indirect association of fathering on bullying victimization, to the authors’ knowledge, no study to date has explored Black father–daughter relationship quality as a mechanism by which involvement and experiences of SBV among girls operate. It is, however, expected that father involvement may increase children’s sense of security, provide unique and supplementary sources of emotional support, and serve as influential prosocial role models [76], thereby mitigating the risk of SBV for their daughters. Thus, we hypothesized that (1) greater father involvement and stronger father–daughter relationship quality will significantly and directly decrease experiences of SBV, and (2) father–daughter relationship quality will significantly mediate the father involvement and SBV relationship. Taken together, Black father involvement and the quality of the father–daughter relationship may be critical in decreasing daughter experiences of SBV in middle childhood.

## 2. Materials and Methods

### 2.1. Participants and Procedures

Data were derived from the Fragile Families and Child Wellbeing Study (FFCWS), a longitudinal U.S. birth cohort study of 4898 focal children and their families. Stratified random sampling was employed, and strata included (1) large cities with a population of 200,000 or more, (2) hospitals within each chosen city, and (3) births identified within the selected hospitals. Mothers and fathers were recruited to participate in the study between 1998 and 2000 following the birth of the focal child, with follow-up periods occurring at focal child aged 1 (wave 2), 3 (wave 3), 5 (wave 4), 9 (wave 5), and 15 (wave 6). Starting at wave five and in subsequent waves, focal children self-reported various factors, including parent, peer, and teacher relations, academic progress and achievement, psychopathology, health, and risk behaviors. Parental consent and child assent were obtained at baseline and subsequent waves, and all study participants received compensation for their participation at each wave (for a detailed description, see [77]).

The current study used de-identified data from the fifth wave of the FFCWS, when the child was 9 years old. Following an approved request from the FFCWS Office of Population, access to the publicly available data was granted. Given the current study’s focus, an analytical sample of n = 368 Black fathers and their daughters (baseline reports of father race and child gender) with complete data on all variables were retained for study analysis. Of girls aged 9 years old (*M* = 9.31, *sd* = 0.39), nearly one-fourth (21.20%) reported being physically bullied, and more than two-thirds (70.66%) reported having “quite to extremely well” relationships with their fathers. Slightly more than half of the fathers lived in/near poverty (52.45%) and reported lower educational attainment (51.9% had ≤high school or equivalent education), and nearly two-thirds of fathers reported not being married to the child’s mother (65.49%).

### 2.2. Measures

#### 2.2.1. Dependent Variable

SBV was measured by the child’s self-reports of how often, in the past month, peers in school and/or their neighborhood excluded and purposely left them out of activities. Children reported on their experiences of SBV using a 5-point Likert response format ranging from 0 “not once in the past month” to 4 “every day”. Given the skewed distribution of the data, responses were dichotomously recoded as never socially bullied (0) and socially bullied (1).

#### 2.2.2. Independent Variables

Father Self-Reported Involvement. Father involvement was assessed by asking how often fathers participated in various activities together with focal child in the past month. Using a Likert scale response of “not in the past month” (0), “1–2 times in the past month” (1), “about once a week” (2), “several times a week” (3), and “every day” (4), fathers self-reported the frequency to which they (1) helped with homework/school assignments, (2) checked to make sure child completed homework, (3) played sports/participated in outdoor activities with the child, (4) did dishes, prepared food, or did other household chores with child, (5) watched TV or videos with child, (6) played video or computer games with the child, (7) read books with/talked with the child about books, (8) participated in indoor activities (i.e., arts and crafts or board games) with child, (9) talked with the child about current events, such as things going on in the news, and (10) talked with the child about her day. The responses for the 10 items were summed to create a father involvement scale, with higher scores indicating greater involvement in the past month. Cronbach’s alpha coefficient demonstrated very good internal consistency (α = 0.876 for our sample).

Father–Child Relationship (Child-Reported). Father–child relationship was assessed using one item gauging the extent to which the father and child talked and exchanged ideas. Derived from the Family Functioning and the Middle Childhood and Adolescent sections of the National Survey of Child Health (NSCH), children self-reported how well they and their father shared ideas or talked about things that really matter using a Likert scale response ranging from “extremely well” (1) to “not very well” (4). Responses were then recoded as “not very well” to “fairly well” (0), “quite well” (1), and “extremely well” (2) to address the skewed distribution of the data (see Table 1).

#### 2.2.3. Covariates

Father’s Education. Parental education is associated with SBV [78]; therefore, the fathers’ self-report of their highest level of educational attainment was examined and controlled for in the model. Response choices included less than high school (1), high school or equivalent (2), some college/tech (3), and college or graduate school (4). To address the skewed distribution of the data, responses were collapsed into a dichotomized variable and recoded to indicate a high school education or less (0) and some college education or more (1).

Father’s Poverty Level. When the child was 9 years old (wave 5), FFCWS constructed poverty ratios based on the U.S. Census Bureau of total household income to poverty thresholds from total household income. Poverty threshold responses included “0–49%” (0), “50–99%” (1), “100–199%” (2), “200–299%” (3), and “300%+” (4). Given the skewed distribution of the poverty level data, response categories were collapsed into “0–199%,” representing those in/near the poverty threshold (0), and “200%+” representing those above the poverty line (1).

Physical Bullying Victimization. Factors related to SBV are also associated with traditional forms of bullying, such as physical victimization. In line with prior research investigating the effects of social and relational forms of victimization [28], physical bullying victimization was controlled for in the study. In this data, children self-reported how often, in the past month, peers in school and/or their neighborhood hit them using a 5-point Likert response format ranging from “not once in the past month” (0) to “every day” (4). Responses were dichotomously recoded as never physically bullied (0) and physically bullied (1).

Father’s Marital Status. Fathers self-reported their marital status to the child’s mother. Response choices included “no, not married to the child’s bio-mom” (0) and “yes, married to the child’s bio-mom” (1).

### 2.3. Analytical Statistical Procedures

An analytical sample with complete data on all variables was retained for study analysis using listwise deletion. A pre-analysis screening was conducted in SPSS v 28 to test analytical assumptions of multicollinearity; correlation coefficients, variance inflation factors (VIF), and tolerance scores revealed that the assumption of multicollinearity was not violated (correlations < 0.70; tolerance statistics ≥ 0.95, and VIFs ≤ 1.06) [79]. Next, a bivariate correlation analysis was performed using SPSS v 28 to determine the relationships between SBV and all variables under study. Given the dichotomous nature of the dependent variable, a binary logistic regression was performed in SPSS v 28 to examine whether Black father involvement and father–daughter relationship quality decreased the likelihood of SBV. Finally, PROCESS macro (version 4.0) for SPSS, a program designed and equipped to conduct mediating models, was used to examine if the mediating role of father–daughter relationship quality explains the underlying mechanisms of the relationship between father involvement and SBV [80]. For the mediating model, unstandardized coefficients were produced in PROCESS and therefore presented along with confidence intervals to indicate indirect model significance.

## 3. Results

### 3.1. Bivariate Correlation Analysis

Bivariate analyses showed that father involvement was not statistically significantly correlated with SBV (*r_s_* = −0.065, *p* > 0.05). Father–daughter relationship quality was statistically significant with SBV (*r_s_* = −0.129, *p* = 0.003). Among covariates, only physical bullying victimization and father’s level of education were statistically significant with SBV. Girls who were physically bullied were more likely to be socially bullied (*r_s_* = 0.282, *p* < 0.001). Girls with fathers who had some college or more education reported less SBV than those with less educated fathers (*r_s_* = −0.106, *p* = 0.018). Father’s poverty level and marital status to the child’s mother were not significantly correlated with SBV (*p* > 0.05). Table 2 displays bivariate correlations for all study variables.

### 3.2. Binary Logistic Regression Analysis

Table 3 reports the results of the binary logistic regression model. Binary logistic regression analysis showed that father involvement was significantly and negatively associated with SBV, in that a one-point increase in father involvement decreased their daughters’ odds of SBV by 3.2% (*OR* = 0.968, *p* = 0.031), controlling for physical bullying victimization, father marital status, and poverty and education level. Father–daughter relationship was also significant with SBV only for those who reported quite well father–child relationships, indicating that girls who reported talking and sharing ideas quite well compared to extremely well with their fathers were 2.8 times more likely to experience SBV, controlling for girls’ report of physical bullying victimization, father education level, poverty, and marital status (*OR* = 2.757, *p* < 0.001). Girls who experienced physical bullying victimization were also five times more likely to experience SBV by their peers compared to their non-physically bullied peers (*OR* = 5.065, *p* < 0.001). Father marital status (*p* > 0.05), poverty level (*p* > 0.05), and educational attainment (*p* > 0.05) were not significantly associated with SBV. The model predicted 72% of the cases correctly and accounted for 19.3% of the variance in social bullying victimization among girls. Regarding the second hypothesis, father–daughter relationship quality was not a significant mediator of the relationship between father involvement and SBV (*B* = −0.0013, (95% C.I. = −0.006–0.002).

## 4. Discussion

This study examined Black father involvement and father–daughter relationships as protective factors in SBV among girls. Results showed that father involvement was significantly associated with lower odds of SBV and support other studies that have found father involvement to be protective in reducing/intervening in unhealthy experiences such as bullying victimization [42,52,54] and other harmful behaviors [14,15,16,17,18,19,20]. In this study child report of the quality of relationship with their father was not significantly associated with SBV for those who reported not-fairly well relationships. However, the quality of relationship with father was significantly associated with a greater likelihood of SBV for those who reported quite well relationships compared to those who reported extremely well relationships. This finding may suggest that whereas quality of the relationship is important, levels of quality also matter. For example, consistency may influence relationship quality, such that more consistency may increase the robustness of the relationship between father and daughter, subsequently decreasing the likelihood of victimization. These findings may also suggest cultural nuances that are not easily measured, in that Black children or children of Black fathers may feel the need to or be encouraged to defend themselves [9], especially in victimizing situations. It is also important to note that in defending themselves, girls may be perceived as bullies rather than victims. Clearly, this study shows that high father involvement and relationship quality matter in navigating and safeguarding their daughters’ social bullying victimization experiences. 

Mediation analyses showed non-significant indirect associations, suggesting that father involvement reduced SBV more directly than indirectly. Finally, regarding covariates, physical bullying victimization was significantly associated with social bullying victimization, as reported by other studies [28]. Considering that SBV is conceptually framed as including direct and indirect manipulation as well as verbal rumors [24], it is not surprising that physical and social bullying victimization are bedfellows, with five times more likelihood of co-occurrence.

It was also interesting that father characteristics such as educational level, socioeconomic status, and marital status did not significantly predict social bullying victimization among this sample, as seen in other studies [78]. While more than half of the fathers lived in/near poverty and reported lower educational attainment, and nearly two-thirds of fathers reported not being married to the child’s mother, it did not change the importance of father–child involvement and its benefit in reducing SBV.

### 4.1. Implications

These findings suggest that father involvement is not only beneficial for children, particularly girls from families of different ethnicities or families with higher SES and educational attainment, but also matters for Black families, families from low SES backgrounds, and families who may have average to below average education. Secondly, father–daughter relationship quality does not necessarily mean that children are opening up to their fathers (and, in extension, primary caregivers) about their experiences of SBV (and probably not about physical bullying victimization as well). Findings also suggest that father involvement cannot be underestimated in the quest to provide protective pillars for child and adolescent development.

It is therefore important to be intentional in increasing father involvement in prevention/intervention programs [8,81]. One way to encourage and normalize the importance of father presence in the lives of children (especially girls) is to talk about such studies in the media to increase awareness to the communities. This is crucial particularly because some studies, including findings from the longitudinal study by Fragile Families and well-being dataset (FFCWS), show that most children born to unwed couples and unstable families are more likely to be Black, in comparison to Hispanic and White families [82]. However, studies also show that Black fathers are more likely to be involved in their children’s lives, especially as non-resident fathers and providers, compared to other ethnic groups [63]. Therefore, promoting the benefits of father involvement regardless of father residency and age is imperative and protective in the long run, especially for daughters.

### 4.2. Limitations and Future Research

While this secondary data set is rich with information, there are limitations to the study that lend themselves to considerations for future studies. First, despite the richness of longitudinal secondary data, there can be limits to what variables can be studied and therefore analyzed based on whether it was measured and at the time point of measurement. Future primary and longitudinal studies should therefore consider including other related parent–child variables to expand and understand parent–child relationship and parent–child involvement measures. For example, father–child relationship quality, closeness, and father–child disclosure to parents are related but different processes and may relate to SBV and other child outcomes differently [43]. Related to this point, only one item captured social bullying victimization in this dataset. Future studies should therefore increase the number of items where possible. Again, this study did not focus on other vulnerable populations, including persons with disability and LGBTQ + individuals. For example, there is data suggesting that children with disability experience higher SBV than peers without disabilities [78]. Further, future studies should consider paying attention to potential cultural nuances when assessing the relationships between father involvement and bullying because differences may emerge given unique group characteristics. In addition, studies should explore what particular involvement activities are more common, effective, and protective for children across ethnicities. Finally, this study focused on girls in middle childhood. Future studies should expand research in this area by examining these hypotheses with younger children and adolescents to understand patterns of Black father involvement, relationship quality, and social bullying victimization over time.

## 5. Conclusions

Findings from this study show that girls who reported quite well father–daughter relationship quality (talking and sharing ideas) than extremely well relationship quality were almost three times more likely to experience SBV, while those who had low relationship quality compared to extremely well relationship quality were not significant. While father involvement is important among fathers, including Black fathers, the absence of/limited father involvement can be detrimental to daughters, including experiencing SBV. Secondly, while father–daughter relationship quality was not a significant mediator of the relationship between father involvement and SBV, both were significantly and independently associated with SBV. To increase awareness of the importance of father involvement and relationship quality, prevention/intervention parent–child programs must be intentional in including fathers, particularly Black fathers, with their daughters. Involvement activities such as doing homework with child, reading with child, cooking, and participating in activities with child are all beneficial and ultimately protective in social situations. This study is also a reminder that a reframe of the narrative and discussion on fathers, particularly Black fathers, is imperative since Black fathers also have many strengths, including parenting and involvement in their children’s well-being.

## Figures and Tables

**Table 1:** Study Descriptive Statistics.

	n (%)	α	M	*sd*
**Child’s Report**				
Social Bullying Victimization				
No	255 (69.29)			
Yes	113 (30.71)			
Father–daughter relationship quality				
Not very to fairly well	108 (29.35)			
Quite well	118 (32.07)			
Extremely Well	142 (38.59)			
Physical Bullying				
No	290 (78.80)			
Yes	78 (21.20)			
**Father’s Report**				
Father Involvement		0.876	21.09	8.87
Poverty				
In/near	193 (52.45)			
Above	175 (47.55)			
Marital Status to child’s mom				
No	241 (65.49)			
Yes	127 (34.51)			
Education				
≤High school	191 (51.90)			
≥Some college or more	177 (48.10)			

Note. n = 368.

**Table 2:** Bivariate Correlation Analyses between all variables.

	1	2	3	4	5	6	7
1. Social Bullying Victimization	1	−0.065	−0.129 **	0.282 **	−0.106 *	0.002	−0.007
2. Father Involvement		1	0.144 **	−0.48	0.134 **	0.030	0.355 **
3. Father–daughter relationship quality			1	−0.156 **	0.115 *	0.114 *	0.197 **
4. Physical bullying victimization				1	−0.157 **	−0.107 *	−0.108
5. Father’s education level					1	0.313 **	0.172 **
6. Father’s poverty level						1	0.153 **
7. Father’s marital status to child’s mother							1

Note. n = 368. * *p* < 0.05; ** *p* < 0.01. Spearman rho coefficients presented.

**Table 3:** Logistic Regression and Mediation Analysis of Father Involvement and Father–Child relationship on Child Social Bullying Victimization.

Independent Variables			
	*OR* [95% CI]	*B* [95% CI]	*p*-Value
Father Involvement	0.968 [0.941–0.997] *		0.031
Father–daughter relationship quality (not fairly well) ^+^	1.639 [0.881–3.050]		0.990
Father–daughter relationship quality (quite well) ^+^	2.757 [1.531–4.964] ***		<0.001
Physical bullying victimization	5.065 [2.861–8.967] ***		<0.001
Father’s education level	0.759 [0.452–1.273]		0.358
Father’s poverty level	1.248 [0.745–2.092]		0.299
Father’s marital status to child’s mother	1.505 [0.847–2.673]		0.110
**Mediating Model**			
Father Involvement → SBV		−0.030 [−0.058–−0.001] *	
Father Involvement → Father–daughter relationship quality		0.005 [−0.005–0.015]	
Father–daughter relationship quality → SBV		−0.267 [−0.563–0.028]	
Indirect pathway		−0.001 [−0.006–0.002]	

Note. n = 368. * *p* < 0.05; *** *p* < 0.001. ^+^ Extremely well is the reference group.

## Data Availability

Data was obtained from the FFCWS Office of Population Research and are available [https://opr.princeton.edu/archive/restricted/Default.aspx (accessed on 8 April 2019)] upon written request, typically in 1 business day.
